# Development of the Windmill Model for Mapping Older Adults’ Intrinsic Capacity Using Digital Twin Technology: Descriptive Qualitative Study

**DOI:** 10.2196/81075

**Published:** 2026-04-13

**Authors:** Yirou Niu, Zehui Xuan, Yanling Wang, Xiaoya Zhang, Yibo Guo, Xin Li, Shuai Jin, Jie Zhao, Qian Xiao, Hong Chang

**Affiliations:** 1 School of Nursing, Capital Medical University, No.10 Xi-tou-tiao,You-an-men Wai Street, Fengtai District, Beijing, 100069, China, 86 17843103693; 2 Department of Neurology, Xuanwu Hospital, Capital Medical University, Beijing, China

**Keywords:** intrinsic capacity, IC, aging, digital twin, DT, avatar, qualitative research

## Abstract

**Background:**

Intrinsic capacity (IC) refers to the sum of the physical and mental capacities of an individual. Conventional IC assessment requires substantial temporal and human resources. Digital twin (DT) technology emerges as a promising solution for efficiently mapping ICs.

**Objective:**

This study aims to explore older adults’ perspectives on the DT technology and their perceptions of how it could effectively represent their ICs.

**Methods:**

A qualitative study was used. Face-to-face semistructured interviews with 23 older adults were conducted. The interviews were transcribed verbatim and analyzed via content analysis approach.

**Results:**

The analysis identified five themes and 16 subthemes: (1) “opt for or not my digital twin,” revealing the older adults’ decisions regarding whether to use DT technology for mapping ICs; (2) “my ideal digital avatar,” describing the older adults’ preferences for personalized digital avatar appearances; (3) “my digital twin maps my intrinsic capacity,” highlighting how multimodal reminders and synchronized avatar changes enhanced their comprehension of ICs; (4) “the benefits my digital twin can deliver,” emphasizing the potential of the DT system to provide feedback services to older adults; (5) “some expectations for my digital twin,” outlining their expectations for DT technology. Based on the above insights, a conceptual model, “windmill” model, was further developed to better understand how to build DTs of older adults and map their ICs.

**Conclusions:**

DT technology was a promising tool for mapping ICs of older adults. Furthermore, the “windmill” model provided a framework to build tailored DTs. The findings of this study could provide references to develop DT model to support IC management.

## Introduction

As the global population continues to age, promoting healthy aging has become an increasingly urgent public health goal. The meta-analysis indicated that only 22% of the global older adults were expected to achieve healthy aging status by 2023 [1]. To address this issue, the World Health Organization (WHO) developed a health care pathway program—integrated care for older people (ICOPE) [2]. The primary focus of ICOPE was to optimize the trajectory of intrinsic capacity (IC) [3]. IC refers to the sum of the physical and mental capacities of an individual, comprising 5 domains, namely cognitive, locomotive, sensory, vitality, and psychological capacities [4]. The prevention of IC decline plays a vital role in promoting healthy aging. The rate of decline in IC of older adults has been found to reach as significant a proportion as 76.1% [5]. Furthermore, it was hypothesized that changes in IC preceded the process of disability development [3]. Therefore, it is essential for screening and assessing IC and implementing interventions to reverse IC decline, with the goal of fostering healthy aging.

Both the first and second versions of the ICOPE framework emphasized the importance of IC assessment [6,7]. IC assessment was typically conducted by health care providers. Specifically, assessment for IC was recommended to be carried out once every 4 to 6 months [8]. However, this approach was time-consuming and labor-intensive. Therefore, the exploration of new assessment tools was warranted. Technological advancements suggest that digital devices might assist in screening and assessing IC [3]. For instance, ICOPE Monitor (IHU Health Age), a digital tool, has been applied to ICOPE implementation [9,10]. Additionally, conversational robots have been used to assist in screening the older adults for IC [8]. Despite these efforts, current digital devices remained limited in their ability to automatically transmit IC-related data, presenting challenges for older adults who faced difficulties in operating digital devices. In contrast, digital twin (DT) technology, with its potential for real-time data integration and predictive analytics [11], might offer a more efficient and comprehensive approach to the screening and assessment of IC.

DT refers to the integration of data from physical models, sensors, and operational history into a multidisciplinary, multiphysical, multiscale, and multiprobabilistic simulation process, which maps the entire lifecycle of the corresponding physical entities in virtual space [12]. The DT facilitated bidirectional data flow between physical and virtual entities to continuously optimize physical counterparts and leveraged historical data to acquire knowledge, predict outcomes, simulate behaviors, and provide valuable insights into the future performance of physical systems [13]. DT has been applied across various fields, including architecture, industry, and aerospace [12]. In recent years, DT has also been applied to the field of health care, demonstrating significant potential for the realization of precision medicine [14]. One representative example is human DT, which can dynamically reflect an individual’s molecular profile, physiological status, emotional and psychological states, and lifestyle evolution [15]. In addition, the study showed DT models could simulate and replicate the wound healing process to facilitate wound management [16]. Within cardiovascular disease, DT technology seeks to predict disease risk and progression in patients and to help clinicians forecast how pathology will evolve against a particular environmental background [17]. Furthermore, the artificial intelligence–enabled DT technology was a lifestyle medicine intervention that results in significant improvements in hypertension [18]. These applications highlighted the versatility and potential of DT in health care.

The 5D DT model can be used to guide the implementation of DT technology. It was proposed in 2018, incorporating physical entities, the model of physical entities in the virtual space, data, services, and connections [19]. This 5D DT model is a general framework that has been applied across various fields [20]. However, existing studies have mainly focused on technical applications and system construction, with limited exploration of how this model can be tailored to represent the IC of older adults. To bridge this gap and adapt the 5D DT model to a health-related framework, this study proposed a preliminary customization based on the IC context. Accordingly, the preliminary design of each component in the customized 5D DT model was structured as follows. First, the physical entity referred to the older adults and their IC levels. Second, the data dimension covered all data related to the IC of older adults, including historical, real-time, and predictive data, involving the collection, storage, management, and analysis processes. Third, services represented the various functions provided within the DT, illustrating how real-world services could be supported and enhanced through digital means. Fourth, connections described the interaction and communication among different components. Finally, the model of physical entities in the virtual space specifically refers to the digital avatar of an older person, designed to accurately reflect his or her level of IC in this study.

This study represents a step toward developing a customized 5D DT model, with a specific focus on exploring the perspectives of older adults. When designing the customized 5D DT model, it is crucial to ensure that the model not only accurately reflects the assessment results of their ICs but also aligns with the self-perception of older adults. Moreover, the DT model must be designed in a way that is both comprehensible and acceptable to older adults, addressing their specific needs and preferences. To translate these theoretical considerations into a practical model, we conducted semistructured face-to-face interviews to explore older adults’ perspectives on the DT concept and their views on how their ICs could be effectively represented within such a model. The findings from these interviews provide the foundational evidence that informs the construction of the customized 5D DT model tailored to map the ICs of older adults.

## Methods

### Study Design

This study used descriptive qualitative research design, which followed the principles of naturalistic inquiry [21]. Face-to-face semistructured interviews with the older adults were conducted between November 2024 and December 2024.

### Study Setting and Participants

Participants were recruited from urban parks and leisure clubs for older adults in Beijing, China, where they engaged in regular recreational and social activities. These sites were chosen because they are popular gathering places for community-dwelling older adults, making them ideal for reaching individuals with diverse levels of ICs. However, we acknowledged that such sampling might introduce a selection bias toward relatively active older adults who are more socially involved. Purposive sampling was used in this study, which enabled researchers to select experienced participants based on the aims of the study [22]. The maximum variation sampling technique was also applied simultaneously to recruit heterogeneous samples of older adults with different levels of ICs, education, sex, and age [23]. The participants had different levels of ICs which were measured by traditional assessment methods (Table S1 in Multimedia Appendix 1), and the educational attainment of participants included primary, secondary, and higher education, to ensure diversity in cognitive and experiential perspectives, offering a wealth of diverse perspectives that could enrich the research topic.

Potential participants were selected according to the following criteria: (1) aged 60 years or older, (2) completed the IC assessment (Table S1 in Multimedia Appendix 1), (3) able to communicate effectively in daily life, and (4) informed consent and voluntary participation in this study.

### Data Collection

Older adults were invited to participate in both the assessments of ICs and qualitative interviews. The IC assessment was conducted using a tablet computer, with specific measurement tools and scoring rules detailed in Table S1 in Multimedia Appendix 1. Interviews were scheduled with individuals who agreed to participate after completing the IC assessment. All participants were assured that their participation was entirely voluntary, and written informed consent was obtained prior to data collection.

Before the interview, the interviewer fully introduced the objective of this study to the older adults. The researcher prepared textual and graphic materials to explain the study to the older adults, helping them understand their ICs and DT technology. In addition, a short video introducing DT technology and the concept of a digital avatar was shown to enhance participants’ comprehension. Everyday examples and scenario-based demonstrations were used to ensure comprehension, and participants were asked to restate their understanding before the discussion began. The older adults were encouraged to actively share their understanding of the applications of the avatar and DT technology, as well as to voice their perspectives and related knowledge during the discussion. Afterward, the older adults were invited to ask any questions they had about the avatar or DT technology, and the researchers provided clear and comprehensive responses to their inquiries. The participants were also told they could withdraw at any point during the interview. The interviews were conducted in a quiet and relaxed cafe environment that allowed for open and comfortable conversation. The interviews were conducted through Chinese conversations. The participants were encouraged to express themselves based on the interview guide. Audio recordings were made with the consent of the participants.

All of the interviews were conducted by the same researcher (YN) to ensure consistency. The researcher was trained by qualitative research experts and practiced interviews to refine their interviewing skills. The interviews were conducted using a semistructured interview guide (Table S2 in Multimedia Appendix 2), which was initially designed based on self-construal theory, providing a deeper understanding of individuals’ self-perception [24]. Then, experts in qualitative research, IC, and DT technology were organized to discuss the interview guide, and the interview guide was revised based on feedback. One participant, who met the inclusion criteria, was selected for a preinterview, and the interview outline was modified based on the results of this preinterview. The data obtained from the preinterview were not included in the analysis. The principle of data saturation was a recognized criterion for determining sample size in qualitative research designs [22]. In this study, data collection and analysis processes were carried out simultaneously, and data collection was stopped when no new data was generated related to the topic of study. Specifically, after interviewing 21 participants, no additional themes related to the study topic were identified, and 2 further interviews were conducted to confirm saturation. Therefore, data collection was stopped once thematic redundancy was observed.

### Data Analysis

The interview recordings were transcribed and organized verbatim within 24 hours of the end of each interview. NVivo 12.0 software (QSR International Pty Ltd) was used for coding and analysis, and data analysis was conducted in Chinese. This study adopted the content analysis approach [25]. The interviewer reviewed the text multiple times to ensure comprehensive understanding of its content. Subsequently, open coding was conducted to identify key ideas and concepts within the material. To ensure comprehensive capture of information, open coding was conducted in a line-by-line, sentence-by-sentence format. Then, similar and related codes were classified into categories and subcategories. Furthermore, themes and subthemes were generated based on categories and subcategories. This process was iteratively repeated until saturation was reached, with no new themes or subthemes emerging, and was independently conducted, confirmed, and discussed by 2 researchers. Finally, the themes and subthemes were translated into English using both direct translation and back-translation methods.

### Rigor

Rigor focuses on 4 key dimensions: credibility, transferability, dependability, and confirmability [26]. The credibility was strengthened by the researcher’s establishment of trusting relationships with older adults, which facilitated authentic self-expression during the interview process. The results of the study were also fed back to the participants, enabling them to confirm and add to the results. Additionally, the researcher used reflexive journaling to track the research process and identify potential biases, thereby enhancing credibility. To enhance transferability, a purposive sampling method was adopted to ensure that participants had rich perspectives. Moreover, rich interview data enhanced transferability by documenting participants’ context, enabling evaluation of results’ applicability to other settings. The dependability was addressed through regular discussions among the researchers. These discussions allowed for the resolution of disagreements and confusion during the analysis process. Finally, the confirmability was established by providing a description and rationale for the decisions made based on the results.

### Ethical Considerations

This study was approved by the ethics committee of the Capital Medical University (batch: Z2023SY138). Informed consent was obtained from all participants, and the study adhered to all relevant privacy and confidentiality protocols. All patient-related information has been fully anonymized and de-identified. No compensation was provided to the participants. All procedures were conducted in accordance with the guidelines of the Institutional Review Board (IRB) and Research Ethics Board (REB).

## Results

### Participant Characteristics

A total of 23 older adults were interviewed face-to-face, with participant selection considering the age, sex, degree of education, and the level of IC. Interviews lasted between 27.75 and 49.58 minutes (mean 37.57, SD 6.77 min). The demographics were outlined in Table 1. Of the 23 older adults, 12 were women and 11 were men, with an average age of 68.30 (SD 5.45; range: 61-84) years. 17 had a high school education or higher, including 6 with a bachelor’s degree or higher. The average score for IC was 4.04 (SD 0.98; range: 2-5).

**Table 1.** Participant information.

Number	Sex	Age (years)	Education level	Chronic diseases	Intrinsic capacity score	Cognition	Psychological capacity	Sensory	Vitality	Locomotive capacity
N1	Male	73	College graduate or higher	0	4	29/normal	1/normal	1/hearing failed	14/normal	12/normal
N2	Female	68	Secondary education level	2	4	28/normal	2/normal	2/normal	14/normal	8/decline
N3	Female	68	Secondary education level	1	5	26/normal	0/normal	2/normal	13/normal	11/normal
N4	Male	67	College graduate or higher	4	5	29/normal	3/normal	2/normal	12/normal	11/normal
N5	Male	64	Secondary education level	1	3	24/decline	4/normal	2/normal	13/normal	7/decline
N6	Female	75	College graduate or higher	1	5	28/normal	2/normal	2/normal	13/normal	11/normal
N7	Female	65	Secondary education level	0	5	29/normal	0/normal	2/normal	13/normal	10/normal
N8	Female	69	Secondary education level	1	5	29/normal	0/normal	2/normal	14/normal	10/normal
N9	Male	65	Secondary education level	3	4	28/normal	9/decline	2/normal	14/normal	12/normal
N10	Male	68	College graduate or higher	0	4	22/decline	2/normal	2/normal	14/normal	11/normal
N11	Female	67	Secondary education level	1	4	29/normal	3/normal	1/vision failed	13/normal	11/normal
N12	Male	73	Secondary education level	1	5	26/normal	2/normal	2/normal	14/normal	12/normal
N13	Female	84	Elementary school	0	2	18/decline	3/normal	0/hearing and vision failed	14/normal	9/decline
N14	Male	61	College graduate or above	1	4	28/normal	3/normal	2/normal	13/normal	4/decline
N15	Male	66	Secondary education level	1	5	28/normal	2/normal	2/normal	14/normal	12/normal
N16	Male	68	Elementary school	0	5	30/normal	0/normal	2/normal	14/normal	12/normal
N17	Male	75	Secondary education level	1	2	23/decline	3/normal	0/hearing and vision failed	13/normal	1/decline
N18	Female	67	Secondary education level	0	4	28/normal	8/decline	2/normal	14/normal	12/normal
N19	Male	75	Secondary education level	2	2	24/decline	6/decline	0/hearing and vision failed	12/normal	11/normal
N20	Female	61	Secondary education level	3	4	30/normal	4/normal	1/vision failed	13/normal	11/normal
N21	Female	61	Secondary education level	1	4	27/normal	4/normal	1/hearing failed	12/normal	11/normal
N22	Female	66	Secondary education level	2	4	28/normal	2/normal	1/vision failed	14/normal	11/normal
N23	Female	65	College graduate or higher	4	4	28/normal	4/normal	0/hearing and vision failed	14/normal	11/normal

### Summary of Themes

#### Overview

The study identified five key themes related to build DTs of older adults to map their ICs: (1) “opt for or not my digital twin,” explored the willingness of older adults to adopt DTs as a means to reflect their ICs; (2) “my ideal digital avatar,” in which older adults depicted the characteristics of their ideal digital avatars; (3) “my digital twin maps my intrinsic capacity,” focused on how DTs could effectively represent and monitor the ICs of older adults; (4) “the benefits my digital twin can deliver,” in which older adults expressed their beliefs regarding the potential benefits that DTs could offer to them; and (5) “some expectations for my digital twin,” expressed the expectations that older adults had for the future development of DTs. From these insights, we further developed a conceptual model to better understand how to build DTs of older adults to map their ICs.

#### Theme 1: “Opt for or Not My Digital Twin”

DT technology, being an emerging concept, was unfamiliar to the majority of older adults. When older adults understood the concept of DT, they were presented with the option to use this technology for monitoring and reflecting on their levels of IC. This decision-making process yielded 4 response outcomes:

##### Subtheme 1: “Health Mirror”—Optional

Some older adults recognized and believed that DTs were optional as health mirrors. They believed that DTs were just like mirrors that could reflect their IC levels and health status. The presentations of DTs were real-time, with changes synchronizing with the IC levels of older adults:

*If one day digital twins could become a reality and come into my life, that would be quite nice, I reckon.*

[N3]

Digital twin is my soul, the soul that represents my physical body and health status. The digital twin is perfectly in sync with me, like two hearts beating as one.

[N9]

##### Subtheme 2: “Multidimensional Advantages”—Worthwhile

Some older adults believed that DTs were well worth choosing because of their multidimensional advantages in terms of convenience, intuitive visibility, scientific rigor, and timely alerts. Moreover, from the perspective of older adults, DTs might be better than physicians. The results generated by DTs were based on real data, which provided a rational basis for accuracy and reliability. However, it was possible for the physicians to selectively disclose certain information to older adults:

*Digital twins can bring a lot of convenience to my life. It can remind you before you are in danger. It’s very useful, isn’t it? My son is not around me, and my health is poor. I am afraid that one day something bad will happen. No one reminds me. No one is around me. It will be much more convenient to have the digital twins*

[N12]

*Doctors, they have their own emotions and thoughts, and mixing them in with the process (clinical care trajectory) is very bad. Digital twins, they have no emotions. They just do what they are supposed to do, and you know what? They are based on data. Well, they are true.*

[N2]

##### Subtheme 3: “Unknown and Unbelievable”—Nonessential

There remained a group of older adults who believed that constructing a DT to reflect their ICs and health status was of little consequence. Many demonstrated limited familiarity with DT and had no practical experience in its use, leading to skepticism or ambivalence toward its adoption. Additionally, a segment of the older adults perceived DT as being overly futuristic and unattainable, particularly within the near-term timeframe. For technologies perceived as infeasible, the question of choice was deemed irrelevant:

*I didn’t know anything about digital twins before, and even now, I’ve only heard a little bit about it. I’ve never actually used it, so I can’t really say much about it. And as for using it? Well, I wouldn’t have a clue how to.*

[N3]

*Well, how should I say it? For someone like me, at this age, even talking about this stuff—digital twin technology—seems like wishful thinking. I don’t waste my time on it because this kind of technology is not going to be ready anytime soon. This thing won’t be around for at least a decade or two. It has nothing to do with me, so whether I choose it or not doesn’t really matter.*

[N10]

##### Subtheme 4: “Proliferation Concerns”—Rejected

Not all older adults actively accepted new technologies, with a subset expressing opposition to the adoption of DTs. They argued that advancements in technology might reduce the need for human labor, leading to job losses or unemployment among younger generations. Without stable employment and income, young people might delay or forgo marriage and childbearing, ultimately contributing to a decline in fertility rates. These adverse outcomes caused by the development of technology could affect human survival and reproduction:

*I surely don’t wish to see such a thing come to pass. If this technology was applied, a few people could take care of all the older adults. What about other young people? How should they survive? Let me tell you this, the more advanced technology becomes, the more people find themselves out of work. But people must have a work and live on. Then they will choose to get married and have children. Why are there so many DINK families coming out? Because technology is too advanced.”*

[N15]

### Theme 2: “My Ideal Digital Avatar”

The appearance of the digital avatar served as the end point for the presentation to older adults and directly affected the effectiveness and adherence of the application. According to the concept of DT technology, the digital avatars were replicas of physical entities that synchronized with the changes in real older adults. Unexpectedly, they voiced some perspectives that were outside the box.

#### Subtheme 5: “Mirror Mapping” and “Visual Refinement”

To better understand their IC and health conditions, some older adults desired the digital avatar to be an exact replica of themselves, reflecting a mirror-image relationship. To avoid negative emotions, such as sadness or self-abasement, that might arise from confronting the realities of aging and poor health, others preferred the digital avatar to embody an idealized, glorified version of themselves. A feasible solution might be to show the real appearance to physicians and a beautified version to the older adults:

*I don’t want the real one. When you look at yourself against this appearance at home, it’s necessary to have an appearance that’s more pleasant to look at. You could design two versions—one that shows the real appearance to the doctors, and another that shows a more comforting and pleasant appearance to us.*

[N1]

#### Subtheme 6: Self-Preference Projection

The ideal digital avatar for older adults varied significantly based on personal preferences. Some chose representations that mirrored themselves, including their current appearance, an enhanced version, or a rejuvenated younger self. Moreover, some older adults said the digital avatar was portrayed as a young person of the opposite sex, which they found more visually appealing and engaging. Others indicated that the digital avatar represented specific organs, enabling them to better understand health conditions. Interestingly, idols, cute pets, and favorite cartoon characters were also regarded as their ideal digital avatar:

*Everyone likes handsome men and beautiful women, don’t they? If you’re a man, of course you’d want a beautiful woman, especially as you get older, right? And for us old ladies, it’s such a delight to see something pleasing to the eye - it just makes us happy!*

[N8]

*Well, if that digital avatar looked like Daoming Chen (an actor), I’d be using it all the time, I tell you! I’d follow every suggestion he makes.*

[N20]

### Theme 3: “My Digital Twin Maps My Intrinsic Capacity”

DT technology can map the ICs of older adults, and the shape and changes of the digital avatar indicate the level and changes in their ICs. The expectations among older adults regarding how DT should reflect the diversity and dynamics of their ICs vary widely.

#### Subtheme 7: Multimodal Presentation

The manifestation of IC variations in DTs of older adults was multimodal, encompassing auditory, visual, animated, and textual modalities. Some older adults indicated that auditory alerts from the DTs were considered an adequate response to IC variations, dimensional changes, or risk factor detection, and visual representations through images or animations were generally well-received among the older adults. Textual presentations, though beneficial for maintaining the privacy of IC results, were not user-friendly for older adults with vision loss:

*My motor function is down. The digital twin of mine is just like the avatar in virtual game—when its energy runs low, it slowly starts to shrink away. It’s like a little nudge, reminding me to get moving and recharge my batteries.*

[N14]

*All I need is for it to show me in plain writing—what’s the test about, what’s the perfect score, how many points I got, what’s considered normal range, and where exactly I stand. Just lay it out simple and clear for me.*

[N18]

#### Subtheme 8: Matching Behavior Mapping

Older adults tend to monitor external changes that align with the 5 dimensions of IC, using these changes as indicators to identify potential issues (Table 2). For example, assistive devices indicated declining motor functions, lateral ear orientation suggested hearing loss, and squinting eyes represented visual impairment. There were still some older adults who said they did not need to have extrinsic changes when their IC levels changed:

*When I can’t move like I used to, my digital twin shows it too—starts walking slower, takes smaller steps, kind of shuffling along like my feet are stuck to the floor.*

[N23]

*Even if my IC decreased, I’d still want my digital twin to look happy and cheerful. You see, when the people around me are smiling and in good spirits, it makes me feel better too. But if they look sick and miserable, it just brings me down.*

[N11]

**Table 2.** Digital avatar’s external changes linked to intrinsic capacity abnormalities.

Dimension and abnormal outcomes	External changes
Cognition	
Cognitive decline	Dull eyes Forgetting behavior Speech delay Look unsure Dementia-like behavior Fixed posture Unresponsive
Psychological capacity	
Mild depressive symptoms	Less communication Moodiness
Severe depressive symptoms	Expression of fear Expression of irritability Expression of anger Expression of giving up Abnormal behavior Social avoidance
Sensory	
Vision failed	Wearing glasses Squinting
Hearing failed	Wearing hearing aids Lean in to listen Unresponsive
Vitality	
Malnutrition or risk of malnutrition	Slim Pale complexion Listless Increased wrinkles Activity reduction
Locomotive capacity	
Decline	With the aid of assistive devices Stride reduction Flustered gait

### Theme 4: “The Benefits My Digital Twin Can Deliver”

From the perspective of older adults, DT offers the following potential benefits.

#### Subtheme 9: Health Advisor

Some older adults regarded DTs as personalized health advisors, capable of enhancing their IC through proactive interventions. Moreover, DTs could perform comprehensive health monitoring, provide early disease detection, support medical treatment, and facilitate health management:

*Every morning when I open my phone and see my digital twin, it’s like getting a little preview of how my day might go. It tells me what I should watch out for, if something’s not quite right with my health, giving me a heads-up before any real trouble starts. It’s like having a little guardian angel in my pocket.*

[N21]

#### Subtheme 10: Life Assistant

Beyond the medical applications, DT was also recognized as a comprehensive life assistant, offering support across various aspects of daily living, such as essential reminders, practical life guidance, and tailored solutions to address the unique challenges faced by seniors living independently:

*In the future, if I am left alone, I will get such a digital twin. It’d be a real help. Like when I feel like cooking, it could show me recipes. And when I want to do my exercises, it could lead me through them.*

[N7]

#### Subtheme 11: Spiritual Mentor

Some older adults not only found joy in engaging with their DTs but also saw them as spiritual mentors in favor of their psychological well-being:

*Sometimes we old folks aren’t too keen on socializing with others. Maybe we don’t quite get along with real people, but chatting with our own digital twin gives us something to do. What’s more, the mental state of my digital twin is exceptionally good, and I consider it to be my role model.*

[N1]

#### Subtheme 12: Role Complement

DT was poised to become an important supplement to the lives of older adults, such as their children, caregivers, or health advisors. For instance, for a group of older adults experiencing geographical separation from their children or those belonging to childless households, DT offered comprehensive solutions by providing both practical assisted living services and essential emotional companionship, thereby effectively supplementing traditional filial support roles:

*My children can’t really take care of me, bless their hearts - they’ve got their own families to look after, and they live so far away. But I’ve got this digital twin of mine, it’s been a real lifesaver. Whether it’s helping me in life or giving me health guidance, it’s always there for me. Honestly, it’s just like having one of my own children by my side.*

[N9]

### Theme 5: “Some Expectations for My Digital Twin”

Older adults expressed some expectations for their DTs, notably the following subthemes.

#### Subtheme 13: Intelligence and Knowledge Integration

The DT technology used to reflect the IC of older adults was expected to be intelligent and knowledge-integrated, enabling automated data transfer, rich functionality, and remote operation. The intelligence and knowledge integration of the DT technology were not only applied in health care, but also extended to various facets of older adults’ daily life, encompassing daily assistance, entertainment, and so on:

*All my information and data just get sent up there automatically—I don't have to bother with typing anything in myself.*

[N10]

*My digital avatar can even dance and sing for me. When I’m in the mood for some Beijing opera, it can belt out those classic tunes too. It’s got all sorts of little entertainment tricks up its sleeve.*

[N13]

#### Subtheme 14: Positive Feedback

Timely positive feedback could help motivate older adults to persist in using DT technology to improve their ICs and health status:

*Every day, my digital avatar gives me exercise guidance. After the workout, it shows me all my vital signs right then and there, making me feel like I’m getting better. Getting this instant feedback really keeps me motivated to stay active and healthy.*

[N16]

#### Subtheme 15: Multisubjective Bond

DTs for older adults were anticipated to function as interconnected systems with multiple entities, serving as bonds that facilitated integration across domestic, community, and hospital networks:

*If the doctor notices my digital avatar ’s head turning red—that means my blood pressure’s up—they can just send me a message right away, telling me what to do about it.*

[N6]

*Let me give you an example—my digital avatar here, it’s not just saw by me, but my spouse too. My son can check in on me anytime, through this system.*

[N14]

#### Subtheme 16: Privacy and Security

The assessment and management of older adults’ IC using DT technology necessitated the huge sensitive personal data. This process should be private and secure:

*The digital twin should have proper security measures. If they go leaking all our private information, we seniors would lose trust in them.*

[N12]

### Conceptual Model

**Figure 1.**
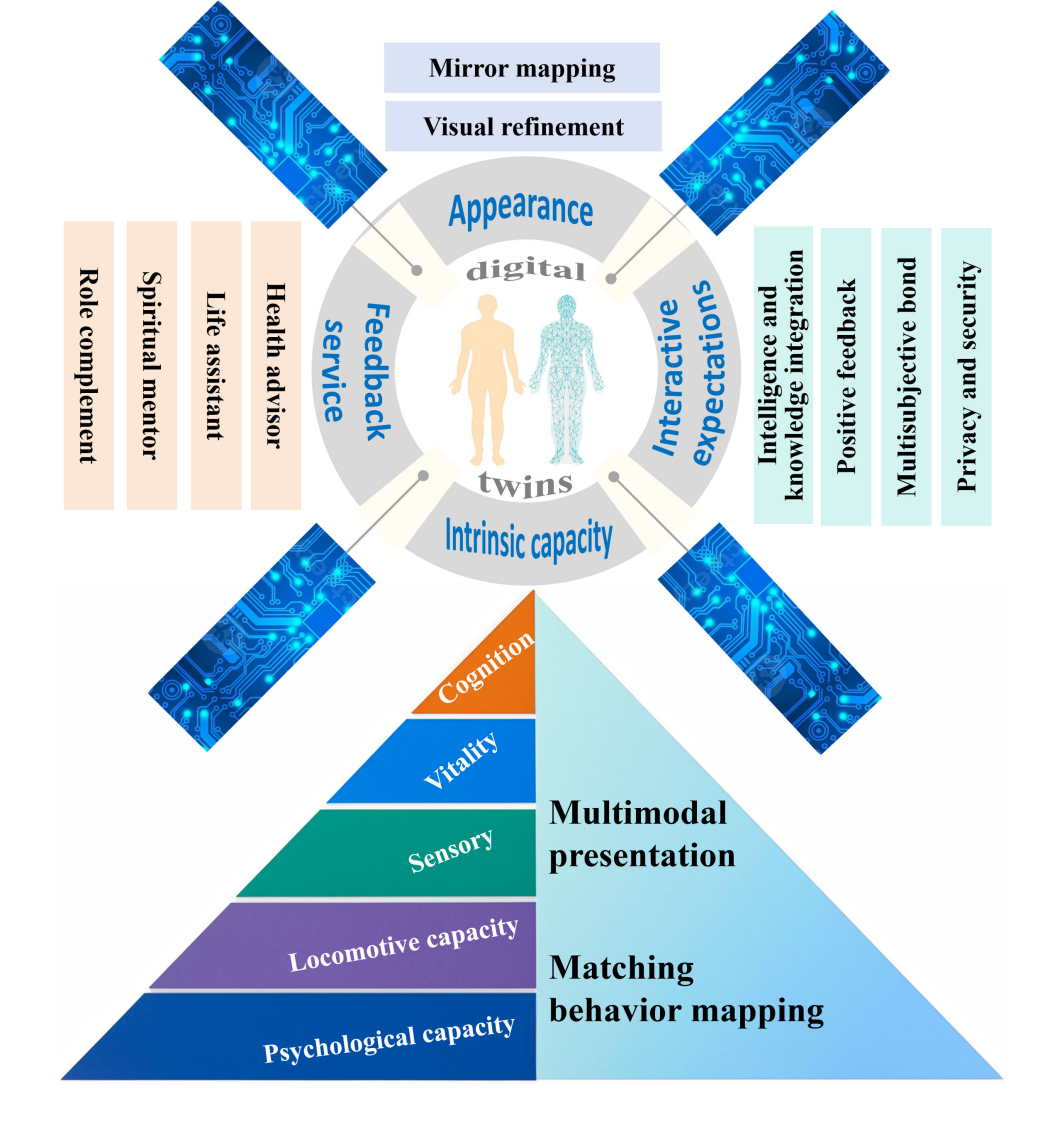
Digital twins focusing on intrinsic capacity of older adults—windmill model.

Based on the insights, we further developed a conceptual model, the “windmill” model, to better understand how to build DTs of older adults to map their ICs (Figure 1). Centered on the evaluation of IC, the “windmill” model integrated DT technology and comprised 4 key components. First, the appearance of the DT, which reflects 2 main preferences: “mirror-like” and “idealized” forms. Second, the mechanism of mapping IC, which enables dynamic visualization of older adults’ IC levels and changes through multimodal presentation and behavioral mapping. Third, feedback service, which includes 4 functional roles: health advisor, life assistant, spiritual mentor, and role supplement. Fourth, interactive expectations, which emphasize intelligence and knowledge integration, the provision of positive feedback, effective coordination among families, communities, and health care institutions, and ensuring the security of personal privacy information. Grounded in the perspective of older adults, this “windmill” model provided support for the application of DT technology in IC assessment.

## Discussion

### Principal Findings

The integration of DT technology into IC assessment held the potential to transform how older adults’ IC was monitored and supported. Incorporating the perspectives of older adults, this study offered an initial exploration of how DT technology might be leveraged to support IC assessment. As for avatar appearance, the participants have demonstrated diverse perspectives regarding their envisioned characteristics of an ideal DT. They also described how the DT should represent and map their IC levels, highlighting the importance of multimodal reminders for enhancing IC comprehension. In addition, participants expected the DT to offer feedback services that could help maintain or improve IC. Based on these findings, a preliminary framework—the “windmill” model—was developed, incorporating 4 core components: appearance, intrinsic capacity, feedback services, and interactive expectations. The “windmill” model offered potential pathways for guiding the design of DT-supported IC assessment tools for older adults.

The appearance of digital avatars played a pivotal role in user engagement and acceptance, which was conducive to helping older adults understand and maintain their ICs. The findings of this study emphasized the importance of personalized digital avatars, consistent with existing literature that indicated superior self-identity recognition with personalized digital representations versus generic avatars [27]. The digital avatars might be one-to-one reductions of physical entities. Individuals might demonstrate an inherent desire to protect avatars that mirrored themselves [28,29], which was beneficial for them to maintain their internal abilities. Digital avatars representing older adults were also expected to take other forms, such as younger versions of themselves, specific organs, or even their idols. It has been shown that whether or not digital avatars resemble the individuals themselves, both can have an impact [30]. Effective interaction, rather than mere personification, might be the key to the success of digital avatars [31]. Therefore, the design of digital avatars should be tailored to the unique characteristics and preferences of older adults to enable effective interactions, rather than strictly replicating their physical appearance.

DT technology realized the dynamic mapping of the ICs of older adults. DT technology could effectively reflect ICs in the following ways: (1) current IC levels and trends, presented through multimodal methods such as voice, text, images, and animations, which cater to diverse user preferences and cognitive abilities; and (2) synchronous changes between digital avatars and the older adults they represent, which were more easily understood. By bridging the gap between complex health data and actionable information, DT technology had the potential to empower older adults to better understand and manage their ICs, which could compensate for the health inequalities caused by the digital divide [32].

The digital avatar had a dual identity—“from avatars to agents” [27]. In this study, digital avatars functioned not only as visual representations to display the ICs of older adults but also as interactive agents capable of providing feedback services. These feedback services focused on health support, life support, and psychological and social support. In terms of health support, digital avatars could provide health status monitoring, early warning, and health education [14]. Additionally, when interacting with digital avatars representing themselves, individuals could adopt an interventionist perspective, leading to a more objective self-assessment [33]. Digital avatars were also expected to provide assistance in daily life for older adults, thereby increasing user engagement and stickiness. Regarding emotional and social support, older adults could identify with their digital avatars, experience meaningful emotions, and form authentic attachments with them. Digital avatars created a beneficial psychological distance that facilitated more objective self-reflection while maintaining emotional engagement [34]. Compared to interactions with real people, individuals perceived digital avatars as more private and secure, making them more willing to express themselves [34,35]. Avatar therapy has been used for the treatment of psychosocial diseases [35-37]. In summary, digital avatars were expected to serve as multifunctional tools.

Older adults expressed some expectations for DT technology in mapping their ICs. First, the process should be intelligent and equipped with sufficient knowledge. In addition to the foundational technologies such as sensors, Internet of Things, machine learning, and deep learning, the intelligence of DT can be further advanced through the integration of multisensor fusion, edge-cloud computing synergy, virtual reality or augmented reality interactive technologies, and generative adversarial networks for data augmentation [38,39]. In the future, DT could be further enhanced by integrating knowledge graphs [40] and expert knowledge [11] to ensure sufficient knowledge support. Second, older adults need positive feedback from DT. Positive feedback has been proven to improve self-efficacy and intrinsic motivation [41]. Third, the DT technology, as a multisubjective bond, connected the older adults, families, communities, and hospitals. From the perspective of older adults, remote health care services become more accessible. For families, it provides a clearer and more comprehensive understanding of older adults’ well-being. Meanwhile, communities and hospitals can achieve batch management of older adults. Finally, the privacy and security of DT technology can be guaranteed by blockchain technology, federated learning, and other methods [11].

Overall, the “windmill” model provided insights from older adults on the construction of the virtual entity, the mapping of IC, the mechanisms of interaction between the virtual entity and his or her twin, and the types of services that a DT system should deliver. It also encompasses the core components of a DT system—the physical entity, its virtual twin, and the mapping relationship linking the two [42]. Compared with the 5D DT model, the “windmill” model offered a distinct theoretical contribution by integrating older adults’ perspectives into the conceptualization of DT-based IC assessment. This user-centered orientation was absent from the 5D DT model, which primarily emphasized system architecture, data flows, and computational mechanisms but offered limited consideration of older adults’ acceptance, perceptions, and needs. However, the “windmill” model presented a notable limitation in that it did not address the data and connectivity dimensions included in the 5D DT model. To enhance its comprehensiveness, future work should incorporate expert consultations to refine these technical dimensions and integrate them with the older adults-centered components.

The IC assessment based on DT technology guided by the “windmill” model showed considerable promise. The IC assessment is grounded in multidimensional data collected from older adults, such as physiological parameters, handgrip strength, and daily activity [43-46]. By integrating general population-level knowledge with real-time individual data from wearable devices, DT technology enabled personalized and continuously refined IC assessments for older adults [47,48]. The entire process of data collection and IC assessment results presentation through DT technology reflects the ongoing transition in modern medicine towards a data-driven paradigm [49]. Beyond assessment, DT technology informed by the “windmill” model also showed strong potential for IC-oriented interventions. The study demonstrated that personalized digital coaching presents a highly promising approach for interventions targeting IC [50]. Virtual entity could also be seen as a form of coaching. The study had shown that a virtual entity equipped with expert-level medical knowledge could serve as a personal physician capable of answering patients’ questions around the clock [51]. In addition, an older adult’s DT could serve as a platform for computer-simulated therapeutic experiments, enabling the design of precisely tailored and personalized treatment plans [51].

However, the application of DT to the IC assessment in older adults still faces several major challenges, such as high implementation costs [52], limited usability for individuals with low digital literacy [53], and ethical concerns regarding data privacy and consent [54]. To overcome these limitations, future DT systems are expected to incorporate generative artificial intelligence–based interfaces to simplify user interaction and adopt low-cost wearable sensors and privacy-preserving data protocols to mitigate financial and ethical constraints [51,55,56]. These technological advances also provide a foundation for practical implementation in real-world settings. Take the Chinese community setting as an example, older adults could wear basic wearable sensors, and the collected data would automatically update their individual DTs. Based on these real-time data streams, the DT model could provide targeted guidance for daily IC management, while community health professionals could remotely assess and manage older adults’ IC through the DT system.

There were several limitations to this study. First, this qualitative study sample involved participants from parks and leisure clubs for older adults but did not include older adults from homes, nursing homes, or hospitals. This limited the generalizability of our findings to the broader population of older adults. Second, all interviewees were from urban areas and had some level of familiarity with DT technology. This might have influenced their perceptions and suggests that the findings might not fully represent older adults living in rural areas or those with limited exposure to DT technology [57]. In the future, a more diverse sample should be considered, including participants from different regions and with varying levels of technological experience, to improve representativeness and capture a wider range of perspectives.

### Conclusions

To integrate DT technology for mapping the ICs of older adults and enable comprehensive management of ICs, a preliminary conceptual model, termed the “windmill” model, was proposed. According to the “windmill” model, the ideal digital avatars of older adults had diverse personalized characteristics. To help older adults understand their ICs, multimodal reminders and synchronous changes of digital avatars were essential. Furthermore, the DT system was believed to provide various feedback services to older adults in terms of health, daily life, psychology, and society. Older adults also had some expectations for DT technology, such as intelligence, knowledge integration, and positive feedback. These findings provided guidance for the development of DT systems tailored to effectively support IC mapping in older adults, ultimately contributing to healthy aging.

## Supplementary material

10.2196/81075 Multimedia Appendix 1 The assessment tools of intrinsic capacity.

10.2196/81075 Multimedia Appendix 2 Semi-structured interview guide summary.

## Abbreviations

DT: digital twin
IC: intrinsic capacity
ICOPE: integrated care for older people
WHO: World Health Organization
