# Supplementary material for: Development of the Windmill Model for Mapping Older Adults’ Intrinsic Capacity Using Digital Twin Technology: Descriptive Qualitative Study
Source: JMIR Aging. 2026 Apr 13;9:e81075. doi: 10.2196/81075 (PMC13075541; doi:10.2196/81075)
Supplement: Multimedia Appendix 1 [file aging-v9-e81075-s001.docx]

Supplementary Table 1**.** The assessment tools of intrinsic capacity.

| **Dimension** | **Tool** | **Scoring rules** |
| --- | --- | --- |
| Cognition | Mini-mental state examination, MMSE | The scale consists of 30 items, with a maximum score of 30 points. Thresholds are set based on educational level: a score of ≤17 for illiterate individuals, <20 for those with primary education, and <24 for those with secondary education or above indicates cognitive impairment; otherwise, cognitive function is considered normal. A decline is scored as 0, and normal is scored as 1 |
| Locomotion | Short physical performance battery, SPPB | The total score is the sum of three test items: the three-position balance test, the gait speed test, and the chair stand test. The maximum score is 12 points. A score of 0-8 indicates impaired physical function, while a score of 9-12 indicates normal locomotion. A decline is scored as 0, and normal is scored as 1 |
| Sensory | Vision: World Health Organization simplified eye examination chart; Hearing: whispered voice test | Vision: if participants can correctly identify any visual target in each row, the test is considered passed; otherwise, it is considered visually impaired. Hearing: a total of three sequences are presented to each ear. Correctly identifying at least two out of three sequences per ear is considered a pass. A score of 1 is assigned if both vision and hearing are normal; otherwise, a score of 0 is assigned |
| Vitality | Short-form Mini-nutritional assessment, MNA-SF | The scale includes 6 items, with a maximum score of 14 points. A score of less than 11 indicates a decline in the vitality, while a score of 11 or above indicates normal vitality. A decline is scored as 0, and normal is scored as 1 |
| Psychology | Geriatric depression scale, GDS-15 | The scale consists of 15 items, with a maximum score of 15 points. A score of 0-4 indicates normal psychological status, while a score of 5-15 indicates a decline in the psychological dimension. A decline is scored as 0, and normal is scored as 1 |

Note: The total score is the sum of scores from five dimensions, with a maximum score of five points.

These tools were digitally presented on a tablet, which the researcher used to evaluate the intrinsic capacities of the elderly. Upon completion of the assessment for each dimension, the score and corresponding level were immediately displayed on the tablet.
