# Supplementary material for: Development of the Windmill Model for Mapping Older Adults’ Intrinsic Capacity Using Digital Twin Technology: Descriptive Qualitative Study
Source: JMIR Aging. 2026 Apr 13;9:e81075. doi: 10.2196/81075 (PMC13075541; doi:10.2196/81075)
Supplement: Multimedia Appendix 2 [file aging-v9-e81075-s002.docx]

Supplementary Table 2**. Semi-structured interview guide summary.**

| **Number** | **Main focus** |
| --- | --- |
| **1** | What are your thoughts on utilizing digital twins as a means of self-representation and as indicators of your intrinsic capacity and health status? |
| **2** | If a digital avatar were designed to represent you, what should it look like? Please describe the digital avatar with as many adjectives as you can, starting with “This digital avatar should be ......” at the beginning of the sentence. |
| **3** | How would you like your digital twin to show your cognitive function? And how should it let you know when your cognitive function change? |
| **4** | How would you like your digital twin to show your mental state? If it's for an older person who's feeling a little sad or very depressed, what do you think the digital twin should look like? |
| **5** | How would you prefer the digital twin to represent your nutritional status? What notification mechanisms should be implemented to alert you when you're at risk of malnutrition or already experiencing nutritional deficiencies? |
| **6** | How should your digital twin show that your eyesight and hearing are good? And if they're not so good, how should it look different to show that? |
| **7** | How should your digital twin show your motor abilities? |
| **8** | Based on the above, do you have any new opinions on the construction of digital twins to reflect your intrinsic capacity? Do you have any expectations for it? |
| **9** | Do you have any other ideas or suggestions to add? |
